# Patterns of Body Shape Diversity and Evolution in Intertidal and Subtidal Lineages of Combtooth Blennies (Blenniidae)

**DOI:** 10.1093/iob/obab004

**Published:** 2021-03-16

**Authors:** Joshua P Egan, Thaddaeus J Buser, Michael D Burns, Andrew M Simons, Peter J Hundt

**Affiliations:** 1 Department of Biological Sciences, Western Michigan University, 2375 West Michigan Ave, Kalamazoo, MI 49006, USA; 2 Department of Fisheries and Wildlife, Oregon State University, 104 Nash Hall, 2820 SW Campus Way, Corvallis, OR 97331, USA; 3 Cornell Lab of Ornithology, Cornell University Museum of Vertebrates, 159 Sapsucker Woods Road, Ithaca, NY 14850, USA; 4 Bell Museum of Natural History, University of Minnesota, 100 Ecology, 1987 Upper Buford Saint Paul, MN 55108, USA; 5 Department of Fisheries, Wildlife and Conservation Biology, University of Minnesota, 2003 Upper Buford Circle, Saint Paul, Minnesota 55108, USA

## Abstract

Marine intertidal zones can be harsher and more dynamic than bordering subtidal zones, with extreme and temporally variable turbulence, water velocity, salinity, temperature, and dissolved oxygen levels. Contrasting environmental conditions and ecological opportunities in subtidal versus intertidal habitats may generate differing patterns of morphological diversity. In this study we used phylogenetic comparative methods, measurements of body length, and two-dimensional landmarks to characterize body shape and size diversity in combtooth blennies (Ovalentaria: Blenniidae) and test for differences in morphological diversity between intertidal, subtidal, and supralittoral zones. We found that subtidal combtooth blennies have significantly higher body shape disparity and occupy a region of morphospace three times larger than intertidal lineages. The intertidal morphospace was almost entirely contained within the subtidal morphospace, showing that intertidal combtooth blennies did not evolve unique body shapes. We found no significant differences in body size disparity between tidal zones, no correlations between body shape and tidal zone or body size and tidal zone, and no body shape convergence associated with tidal zone. Our findings suggest that a subset of combtooth blenny body shapes are suitable for life in both subtidal and intertidal habitats. Many species in regions of morphospace unique to subtidal combtooth blennies exhibit distinct microhabitat use, which suggests subtidal environments promoted morphological diversification via evolutionary microhabitat transitions. In contrast, limited intertidal body shape diversity may be due to strong selective pressures that constrained body shape evolution and environmental filtering that prevented colonization of intertidal zones by certain subtidal body shapes.

## Introduction

Evolutionary habitat transitions can shape phenotypic evolution and generate disparate patterns of morphological diversity among even closely related clades (McGuigan et al. 2003; Egan et al. 2018; Tavera et al. 2018; Kolmann et al. 2020; Maile et al. 2020). Habitats that offer ecological opportunities can promote morphological diversification, leading to the evolution of novel morphologies and high morphological diversity within clades (Price et al. 2013; Arbour and López-Fernández 2013; Des Roches et al. 2015; Ribeiro et al. 2018). Morphological diversification may be hindered in habitats with few ecological opportunities or that impose strong selective pressures that drive morphological evolution toward a single or small number of adaptive optima (de Alencar et al. 2017). In such cases, clades may evolve novel, habitat-specific morphologies, while simultaneously exhibiting limited interspecific morphological diversity (de Alencar et al. 2017; Sansalone et al. 2019).

In marine environments, one of the most striking habitat transitions occurs between intertidal (above water during low tide and underwater at high tide) and subtidal zones (permanently submerged). Marine intertidal zones are generally harsher and more dynamic than adjacent subtidal habitats, with extreme and temporally variable turbulence, water velocity, salinity, temperature, pH, and dissolved oxygen (Shenker and Dean 1979; Davenport and Woolmington 1981; Menge and Lubchenco 1981; Leigh et al. 1987; Castellanos-Galindo et al. 2005; Mandic et al. 2009). For example, water velocities in rocky intertidal zones can be as much as two orders of magnitude greater than bordering subtidal zones (Gaylord 1999). Contrasting environmental conditions and ecological opportunities in subtidal versus intertidal habitats may generate contrasting patterns of morphological diversity. The larger area and greater depth range of subtidal habitats might provide more ecological opportunities than intertidal habitats, resulting in greater subtidal morphological diversity. For example, benthic to pelagic habitat transitions within subtidal environments have been linked to morphological diversification (Ribeiro et al. 2018; Tavera et al. 2018; Maile et al. 2020; Rincon-Sandoval et al. 2020). The dynamic and harsh conditions in intertidal zones may drive the evolution of novel morphologies not found in subtidal biotic communities, but also limit morphological diversity by driving morphological evolution towards a small number of adaptive optima. In addition, morphological diversity might be limited if organisms with morphologies poorly adapted to intertidal habitats are prevented from colonizing intertidal zones (i.e., environmental filtering; Horn et al. 1999; Kotrschal 1999; Boyle and Horn 2006). However, it is also possible that attributes of intertidal zones, such as reduced aquatic predation pressure or structural complexity, might foster morphological diversification (Horn et al. 1999; Ord et al*.* 2017).

Although subtidal–intertidal evolutionary transitions are relatively rare, multiple lineages of ray-finned fishes have successfully colonized the intertidal, including sculpins (Cottidae), surfperches (Embiotocidae), gunnels (Pholidae), and combtooth blennies (Blenniidae; Kotrschal 1988, 1989; Horn et al. 1999; Kotrschal 1999; Boyle and Horn 2006; Knope and Scales 2013; Soares et al. 2013; Hundt et al. 2014a). In fishes, colonization of intertidal environments has been hypothesized to drive the evolution of morphologies distinct from subtidal species that enable use of crevices and holes in rocks and maintenance of positions on or near the substratum in shallow, turbulent, high-flow environments, such as streamlined, cylindrical, or dorsoventrally compressed body shapes, small body sizes, and large, ventrally positioned pectoral and pelvic fins (Kotrschal 1988, 1989, 1999; Martin 1995; Horn 1999; Boyle and Horn 2006; Soares et al. 2013). However, hypotheses about morphological evolution in intertidal zones are primarily informed by qualitative observations of intertidal fish morphology and studies testing for correlations between body shape evolution and water flow in freshwater systems, which may be poor analogs for intertidal environments (Kerfoot and Schaefer 2006; Meyers and Belk 2014; Natsumeda et al. 2014). Most studies that examined differences in subtidal and intertidal fish morphology did not account for phylogenetic relationships in statistical analyses and many of those that did lacked the phylogenetic replication necessary to draw strong conclusions about morphological evolution in the intertidal (Knope and Scales 2013; Buser et al. 2017). One notable exception is a phylogenetic comparative study by Ord and Hundt (2020) that discovered intertidal combtooth blennies did not evolve smaller body sizes than subtidal species. Additional phylogenetic comparative research is needed to determine if the morphologies of intertidal and subtidal fishes are distinct and if intertidal environments promote or impede morphological evolution.

Combtooth blennies are an excellent clade for studying morphological evolution associated with intertidal habitats. This family of small fishes (most < 10 cm standard length [SL]) contains ∼387 species that can be found from tropical to moderately temperate latitudes and in marine subtidal, intertidal, and supralittoral (area above high tide line that is splashed, but not submerged) habitats with a few species occurring in freshwater (Hastings and Springer 2009). Combtooth Bennies exhibit substantial size and shape diversity. Although many species do not achieve lengths longer than 10 cm SL, some species grow much larger, including the Giant Blenny (*Scarthichthys gigas*) and the Hairtail Blenny (*Xiphasia setifer*), which have maximum reported SLs of 22.2 cm and 53.0 cm, respectively (Froese and Pauly 2020). Some species are elongated (e.g., *Plagiotremus* spp. and *Xiphasia* spp.) and others are short and deep-bodied (e.g., *Pereulixia kosiensis*; Froese and Pauly 2020). Combtooth blennies transitioned to intertidal habitats at least four times and out of intertidal habitats at least seven times (Hundt et al. 2014a) and recent molecular studies have improved our understanding of combtooth blenny systematics (Lin and Hastings 2013; Hundt et al. 2014a; Gibbs et al. 2018; Hundt and Simons 2018). After the larval stage, most intertidal combtooth blennies have small home ranges and rarely leave intertidal habitats (i.e., are intertidal residents; Thomson and Lehner 1976; Wilson 2001; Duci et al. 2009). The phylogenetic replication of subtidal–intertidal transitions within combtooth blennies and progress in systematics provides a framework for conducting robust statistical tests of hypotheses about tidal zone evolution using phylogenetic comparative methods (Felsenstein and Felsenstein 2004; Maddison and Fitzjohn 2015).

The objectives of this study were to quantify combtooth blenny body shape and size diversity and test for differences in morphological diversity between intertidal, subtidal, and supralittoral zones. To accomplish our objectives we: (1) used geometric morphometric techniques (reviewed in Rohlf and Marcus 1993; Adams et al. 2004, 2013) to describe combtooth blenny body shapes using two-dimensional (2D) landmarks on photographs of preserved museum specimens; (2) estimated the evolutionary history of tidal zone use (freshwater, subtidal, intertidal, and supralittoral); (3) characterized patterns of combtooth blenny body shape evolution by plotting the distribution of blenny taxa in principal component (PC) phylomorphospace; and (4) used phylogenetic comparative methods to test for differences in patterns of body shape and size evolution between tidal zones.

## Materials and methods

### Phylogeny and taxon sampling

We conducted all statistical analyses in the R programming environment (R v3.4.0; R Core Team 2017), unless stated otherwise. This study used the maximum clade credibility combtooth blenny phylogenetic tree from Hundt and Simons (2018) based on concatenated Bayesian analyses of the sequences of five nuclear exons (*ENC1*, *myh6*, *ptr*, *tbr1*, and *sreb2*). For comparative analyses we trimmed taxa with missing ecological and morphological character data from the phylogeny using the *drop.tip* function in the “ape” package (Paradis et al. 2004) resulting in a tree for body shape analyses containing 71 species (1 freshwater, 42 intertidal, 26 subtidal, and 2 supralittoral species) representing all major combtooth blenny lineages. Museum catalog numbers and tidal zone character data for all specimens used in this study are in Table 1.

**Table 1:** Museum catalog numbers (Catalog #), tidal zone character states (Tidal zone), and SL (mm) for all specimens used in this study

Species	Catalog #	SL (mm)	Tidal zone
*Aidablennius sphynx*	uncataloged	36.00	intertidal
*Alticus arnoldorum*	JFBM-46349	62.33	supralittoral
*Andamia tetradactylus*	JFBM-47821	84.66	supralittoral
*Antennablennius bifilum*	SAIAB-55369	54.80	intertidal
*Blenniella bilitonensis*	JFBM-47077	86.17	intertidal
*Blenniella chrysospilos*	JFBM-46372	57.87	subtidal
*Blenniella paula*	JFBM-46402	72.35	subtidal
*Blenniella periophthalmus*	JFBM-47847	76.36	intertidal
*Blennius ocellaris*	JFBM-47167	84.18	subtidal
*Chasmodes bosquianus*	JFBM-46472	44.16	intertidal
*Cirripectes castaneus*	JFBM-47857	49.25	subtidal
*Cirripectes polyzona*	JFBM-46374	44.16	subtidal
*Cirripectes variolosus*	JFBM-19178	57.66	subtidal
*Cirrisalarias bunares*	KAUM-I38350	27.33	intertidal
*Crossosalarias macrospilus*	JFBM-47286	47.21	subtidal
*Ecsenius bicolor*	JFBM-46381	45.73	subtidal
*Ecsenius lineatus*	JFBM-47801	66.14	subtidal
*Ecsenius namiyei*	JFBM-47001	81.21	subtidal
*Ecsenius opsifrontalis*	JFBM-46380	27.25	subtidal
*Ecsenius yaeyamaensis*	JFBM-47024	46.06	subtidal
*Enchelyurus kraussii*	JFBM-46759	27.95	subtidal
*Entomacrodus decussatus*	JFBM-47844	104.13	intertidal
*Entomacrodus nigricans*	JFBM-20535	61.64	intertidal
*Entomacrodus niuafoouensis*	JFBM-46266	45.33	intertidal
*Entomacrodus sealei*	JFBM-46267	25.20	intertidal
*Entomacrodus stellifer*	JFBM-47149	55.60	intertidal
*Entomacrodus striatus*	JFBM-46350	58.44	intertidal
*Exallias brevis*	JFBM-46766	57.04	subtidal
*Hypsoblennius hentz*	JFBM-46471	90.46	intertidal
*Istiblennius dussumieri*	JFBM-47798	77.99	intertidal
*Istiblennius edentulus*	JFBM-46743	102.18	intertidal
*Istiblennius lineatus*	JFBM-47101	84.69	intertidal
*Lipophrys pholis*	MNHN-2012-0225	111.15	intertidal
*Lipophrys trigloides*	MNHN-2012-0249	82.00	intertidal
*Meiacanthus atrodorsalis*	JFBM-46386	43.96	subtidal
*Meiacanthus kamoharai*	JFBM-47002	80.37	subtidal
*Microlipophrys canevae*	MNHN-2012-0222	48.00	intertidal
*Microlipophrys dalmatinus*	JFBM-47165	26.18	intertidal
*Nannosalarias nativitatis*	JFBM-46732	39.35	intertidal
*Omobranchus anolius*	USNM-197621	46.90	intertidal
*Omobranchus banditus*	JFBM-37501	53.02	intertidal
*Omobranchus elegans*	JFBM-47136	58.46	intertidal
*Omobranchus fasciolatoceps*	JFBM-47139	51.68	intertidal
*Omobranchus longispinis*	JFBM-46756	33.50	subtidal
*Omobranchus obliquus*	JFBM-46842	33.93	intertidal
*Omobranchus punctatus*	JFBM-47135	83.15	intertidal
*Ophioblennius macclurei*	JFBM-46840	54.97	subtidal
*Parablennius gattorugine*	MNHN-2012-0229	139.00	intertidal
*Parablennius incognitus*	MNHN-2012-0236	35.00	intertidal
*Parablennius parvicornis*	MNHN-2012-0238	149.00	intertidal
*Parablennius pilicornis*	MNHN-2012-0239	69.00	subtidal
*Parablennius rouxi*	MNHN-2012-0242	66.00	subtidal
*Parablennius ruber*	MNHN-2012-0243	95.00	intertidal
*Parablennius sanguinolentus*	MNHN-2012-0246	104.00	intertidal
*Parablennius zvonimiri*	MNHN-2012-0248	34.50	intertidal
*Petroscirtes breviceps*	JFBM-47141	51.49	subtidal
*Petroscirtes mitratus*	JFBM-46362	50.44	subtidal
*Plagiotremus rhinorhynchos*	JFBM-46722	78.48	subtidal
*Plagiotremus tapeinosoma*	JFBM-46762	61.94	subtidal
*Praealticus margaritarius*	JFBM-46729	56.29	intertidal
*Praealticus poptae*	JFBM-46352	44.88	intertidal
*Praealticus tanegasimae*	JFBM-47102	86.15	intertidal
*Rhabdoblennius nitidus*	JFBM-47143	56.23	intertidal
*Rhabdoblennius snowi*	JFBM-46264	23.35	intertidal
*Salaria fluviatilis*	uncataloged	68.59	freshwater
*Salarias holomelas*	JFBM-47013	50.50	subtidal
*Salarias sinuosus*	KAUMI38385	42.40	intertidal
*Scartella cristata*	JFBM-46254	58.45	intertidal
*Scartella emarginata*	JFBM-47147	45.90	intertidal
*Scartichthys viridis*	JFBM-46846	52.44	intertidal
*Xiphasia setifer*	JFBM-46993	473.93	subtidal

Museum abbreviations associated with catalog numbers are defined in Sabaj (2019).

### Characterization of body shape via 2D landmarks

**Fig. 1:**
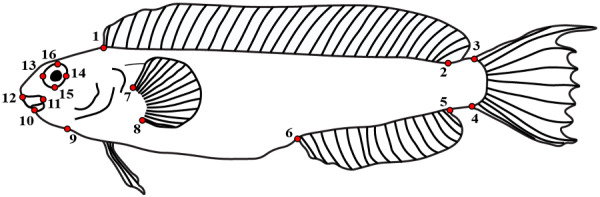
2D line drawing of *Meiacanthus kamoharai* (KAUM-I 38386) in lateral view showing landmarks used for shape analyses: (1) insertion of the most anterior ray of the dorsal fin, (2) insertion of the most posterior ray of the dorsal fin, (3) dorsal insertion of the caudal fin, (4) ventral insertion of the caudal fin, (5) insertion of the most posterior ray of the anal fin, (6) insertion of the most anterior ray of the anal fin, (7) dorsal insertion of the pectoral fin, (8) ventral insertion of the pectoral fin, (9) isthmus of branchiostegal membrane, (10) anterior-most tip of dentary, (11) dorsoposterior-most tip of maxilla, (12) anterior-most tip of premaxilla, (13), most anterior point of eye, (14) most posterior point of eye, (15) most ventral point of eye, and (16) most dorsal point of eye.

To quantify body shape, we first photographed the left lateral aspect of preserved museum specimens using the Pérez (2009) phototank method, with a sample size of one individual per species. All fishes included in this study are bilaterally symmetric, and the body shape of bilaterally symmetric fishes has been quantified from 2D images of the left lateral aspect in a wide variety of species, for example, cichlids (Cichlidae; Kerschbaumer and Sturmbauer 2011), sticklebacks (Gasterosteidae; Walker and Bell 2000), pacus and piranhas (Serrasalmidae; Huie et al. 2019), and sea basses and groupers (Serranidae; Cavalcanti et al. 1999), including species that are morphologically and ecologically similar to combtooth blennies, such as intertidal sculpins (Cottoidea; Buser et al. 2017). Using photographs of each specimen, we recorded the position of 16 external landmarks that are present in all species in our dataset using tps-Dig2.2 (Rohlf 2007; Fig. 1, see caption for landmark location descriptions). The landmarks are adapted from those described in seminal studies of fish body shape using morphometrics (e.g., Strauss and Bookstein 1982; Strauss and Fuiman 1985) as well as from geometric morphometric studies of the body shape of taxa morphologically similar to combtooth blennies, such as sculpins (Buser et al. 2017) and gobies (Cerwenka et al. 2014). We selected the landmark locations used in the present study to capture variation in head and mouth shape (eight landmarks) and postcranial body shape (eight landmarks; see Fig. 1). The ratio of cranial to postcranial landmark locations in this study is consistent with other geometric morphometrics studies of body shape in fishes (Claverie and Wainwright 2014; Cerwenka et al. 2014; Buser et al. 2017).

### Tidal zone and SL character data

We used discrete tidal zone coding from Hundt et al. (2014a; subtidal, intertidal, supralittoral, or freshwater), in which species that occur in both subtidal and intertidal habitats were coded as intertidal. We obtained the maximum reported SL for each species from scientific articles (Springer 1967; Lal Mohan 1968; Springer and Smith-Vaniz 1968; Smith-Vaniz 1971, 1976, 1980; Springer 1972, 1976, 1988; Springer and Gomon 1975; Springer and Spreitzer 1977; Williams 1990, 1988; Carlson 1992; Springer and Williams 1994; Neat and Locatello 2002; Bath 2008; İlkyaz et al*.* 2008; Rangel and Mendes 2009; Hundt et al. 2014a,b; Platt et al. 2016) and FishBase (Froese and Pauly 2020).

### Evolution of tidal zone use

We conducted an ancestral state reconstruction of tidal zone using our 71 species dataset using the maximum likelihood with a Markov k-state 1 parameter (Mk1) model of evolution (Lewis 2001) in Mesquite v3.51 (Maddison and Maddison 2018) to determine the number of habitat transitions represented in our dataset and to visualize our data in phylomorphospace. Hundt et al. (2014a) estimated the evolutionary history of tidal zone use in combtooth blennies with the same phylogeny used by the present study and provided a thorough discussion of combtooth blenny tidal zone use evolution. Therefore, the present study does not discuss this topic in detail.

### Blenny body shape variation

To minimize the effects of nonshape variation (e.g., size, rotation) in our data, we aligned the landmark arrays of each specimen using Procrustes superimposition (i.e., General Procrustes Analysis, see Rohlf and Slice 1990; Zelditch et al. 2012) with the function gpagen from package geomorph v.3.0.4 (Adams and Otárola-Castillo 2013; Adams et al. 2016). Landmark data and an annotated R script that performs all operations conducted in R is available in Supplementary Information 1.

To describe major trends in body shape variation in our dataset, we performed a PC analysis (PCA) on the shape data (i.e., Procrustes-aligned landmark coordinates). We visualized the shape changes associated with the PC axes and the distribution of our taxa in PC morphospace using functions in the packages geomorph, phytools v.0.6-20 (Revell 2012), shapes v.1.2.0 (Dryden 2017), vegan v.2.4.3 (Oksanen et al. 2017), and geiger v.2.0.6 (Harmon et al. 2008). We inferred the evolutionary history of shape change in this space following the phylomorphospace approach of Sidlauskas (2008).

We visualized shape variation for the first four PC axes using Thin Plate Spline methods (Bookstein 1989; Klingenberg 2013). We produced deformation grids and warped outlines of body shape using functions from geomorph to visualize how the PC loadings influenced body shape for each PC. For the latter, we warped an outline illustration of *Scartella cristata* (JFBM 46254) to take on the mean shape of our dataset (i.e., the shape represented by the mean values of each landmark from our dataset). We warped this mean shape outline to take on the shape of the most extreme values observed for each PC axis. This effectively isolates the variance in shape captured by each PC axis and illustrates differences from the mean. See Rohlf (1998) for further discussion of this method of visualizing shape change.

Preliminary analyses identified an outlier in phylomorphospace: *X. setifer*. The body of this species is extraordinarily elongate largest specimen 53.2 cm SL (Smith-Vaniz 1976) compared to the rest of the taxa analyzed herein (most <15 cm SL). Since it is possible for a unique species to influence results, we re-ran all analyses, including Procrustes superimposition, with *X. setifer* removed from datasets for comparison.

### Patterns of body shape and size evolution

We used five approaches to identify differences in body shape and size evolution between tidal zones in combtooth blennies: (1) visual inspection of blenny body shape phylomorphospace plots; (2) pairwise disparity tests for differences in body shape and size disparity between tidal zones; (3) phylogenetic multivariate analysis of variance (phylogenetic MANOVA) tests for correlation between tidal zone and body shape; (4) phylogenetic analysis of variance (phylogenetic ANOVA) tests for correlation between tidal zone and body size; and (5) multivariate convergence tests (*C* tests) testing for convergent evolution of body shape associated with tidal zone (i.e., do lineages that independently colonize a tidal zone evolve into a restricted region of morphospace distinct from ancestors and relatives; Stayton 2015). We tested for differences in body shape and size disparity between habitats by comparing the Procrustes variances of landmark coordinates (for body shape) and maximum reported SLs (for body size) of species in each habitat using the morphol.disparity function from geomorph. The significance of variation between groups was assessed statistically using a permutation technique to generate a null distribution by randomizing shape matrix rows relative to group assignment 1000 times (Adams and Otárola-Castillo 2013). To identify correlations between tidal zone and body shape, we conducted phylogenetic MANOVA, with the Procrustes-aligned landmark coordinates as our independent variables and tidal zone as our dependent variable using the procD.pgls function from geomorph, as well as various helper functions from caper v0.5.2 (Orme et al. 2013) and nlme v3.1-131 (Pinheiro et al. 2015). To identify correlations between tidal zone and body size, we conducted phylogenetic ANOVA using with phylANOVA function from phytools using the maximum reported SL of each species as the independent variable and tidal zone as dependent variable. Convergence tests calculate convergence measures (*C* measures) *C*_1_, *C*_2_, *C*_3_, and *C*_4_, then assess their statistical significance by generating null distributions via simulation (Stayton 2015). Each *C* measure estimates the extent of phenotypic convergence by calculating maximum and contemporary phenotypic distances between focal lineages, then quantifying reductions in phenotypic disparity among lineages through time relative to their maximum phenotypic distance. Convergence measures *C*_1_–*C*_4_ differ slightly in how they quantify reductions in phenotypic distance among lineages (reviewed by Stayton 2015). The *C* measures accommodate multivariate phenotypic data (e.g., multiple PC axes) and values range from 0 (no convergence) to 1 (strong convergence). We calculated *C*_1_, *C*_2_, *C*_3_, and *C*_4_ using combtooth blenny shape data (PC1–PC4) with the convrat function and assessed significance with the convratsig function (500 iterations) in the convevol package (Stayton 2015).

## Results

### Evolution of tidal zone use

**Fig. 2:**
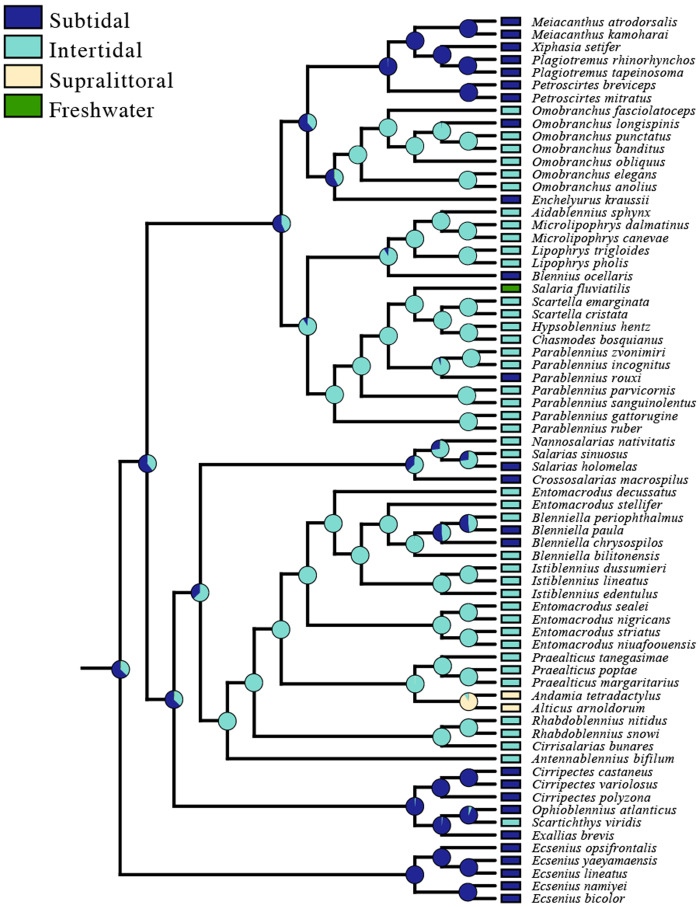
Ancestral state reconstruction of tidal zone in combtooth blennies estimated using the maximum likelihood with a Markov k-state 1 paramter (Mk1) model of evolution in Mesquite. A subtidal character state was inferred to be the ancestral condition of combtooth blennies.

Ancestral state reconstructions found subtidal habitat use as the ancestral state for combtooth blennies. There were four transitions from subtidal to intertidal, seven transitions from intertidal to subtidal, one transition from intertidal to freshwater, and one transition from intertidal to supralittoral habitats (Fig. 2).

### PC analyses

**Fig. 3:**
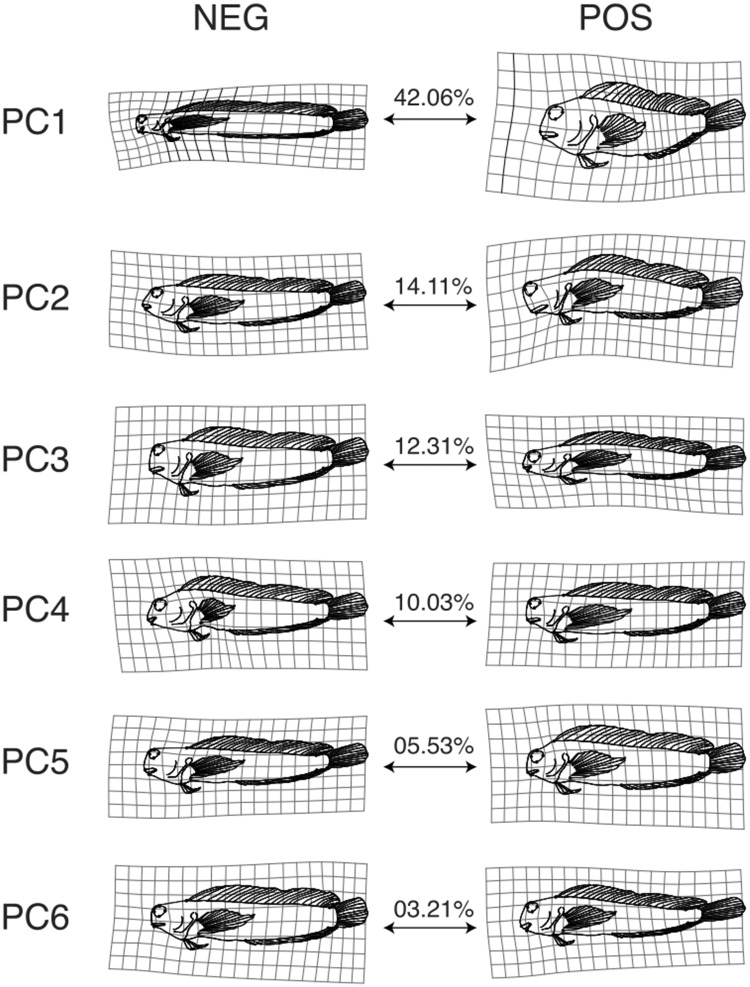
The shape variation described by each of the first six PC axes. Deformation grids and line drawings depict the average combtooth blenny body shape warped to take on the shape captured by each of the extreme ends of each PC axis. The percent of total variance represented by each axis is indicated between the two extreme shapes.

**Fig. 4:**
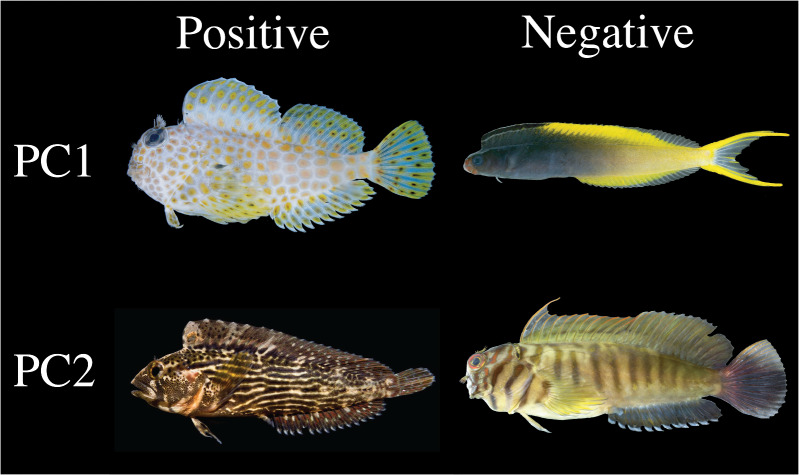
Species representing the extremes of PC axes 1 and 2: PC1+ (*Exallias brevis*; KAUM-I 90009; 32.2 mm SL), PC2+ (*Chasmodes bosquianus*; 44.2 mm SL), PC1− (*Plagiotremus laudandus*; KAUM-I 37778; 51.6 mm SL), and PC2− (*Cirripectes castaneus*; KAUM-I 38357; 64.7 mm SL). Photographs of *E. brevis*, *P. laudandus*, and *C. castaneus* by H. Motomura. Photograph of *C. bosquianus* by J. Bissette.

The first four components accounted for ∼80% of variance in the body shape dataset. In all but the first PC axis, the presence of *X. setifer* had a limited effect (<2%) on the percent of variation captured by PC axes. The variation in shape captured the first six PC axes is presented in Fig. 3 (see Supplementary Table S1 for the PC loadings of each landmark). Together, the first six axes account for ∼89% of the shape variance in our dataset and the remaining 22 PC axes each account for <3% of the total variance. The R-script in Supplementary Information 1 can be used to visualize PC axes. The primary axis of shape variation (PC1) is characterized by dorsal–ventral compression/expansion of the head and body, anterior–posterior shortening/lengthening of the cranial region, anterior–posterior shortening/lengthening of the postcranial region, and a gradient of orientations of the mouth, such that dorsal–ventral compression of the body accompanies anterior–posterior lengthening of the head and an inferior placement of the mouth (Figs. 3 and 4). This axis describes ∼50% of the observed shape variance in the full dataset and ∼42% of the variance in the dataset in which *X. setifer* was removed (hereafter referred to as the “reduced dataset”). The secondary axis of shape variation (PC2) describes ∼14% of the variance of the full dataset and ∼12% of the variance of the reduced dataset. This axis captures dorsal–ventral compression/expansion of the head and, to a lesser degree, the body. This axis also captures anterior–posterior shortening/lengthening of the mouth, and the placement of the eyes, such that dorsal–ventral expansion of the head and body is associated with anterior–posterior lengthening of the mouth and anterior–dorsal placement of the eyes (Figs. 3 and 4). The tertiary axis of shape variation (PC3) describes ∼11% of the variance of the full dataset and ∼12% of the variance of the reduced dataset. This axis captures dorsal–ventral compression/expansion of the body and the head. The quaternary axis of shape variation (PC4) describes ∼8% of the variance of the full dataset and ∼10% of the variance of the reduced dataset. This axis captures variation in the placement of the orbit relative to the rest of the head and body along with lengthening or shortening of the snout relative to the rest of the body.

### Patterns of body shape and size evolution

**Fig. 5:**
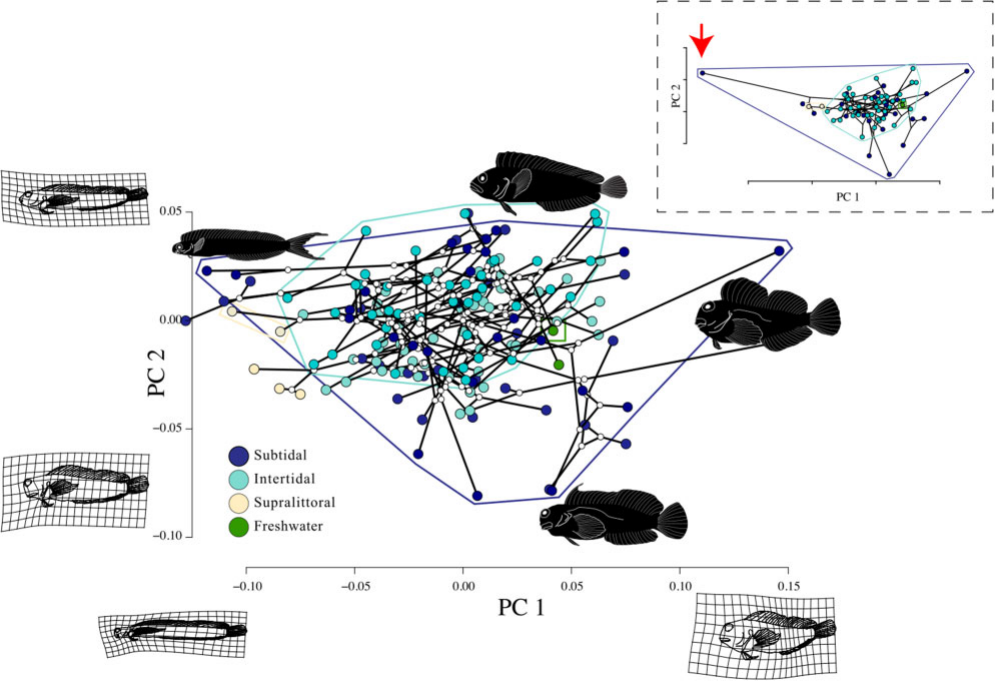
Combtooth blenny phylomorphospace plot depicting the evolution of blenny tidal zone use and body shape with PC axis 1 on the x axis and PC axis 2 on the y axis. At the ends of plot axes, the shape variation described by each PC axis is shown with deformation grids and line drawings depicting the average blenny body shape, which was warped to represent high and low extreme value of each axis. Polygons (convex hulls) surround the species found in a given habitat and the color of the polygon outline matches that of the habitat that it represents. Line drawings next to the tips of the phylomorphospace plot depict the species with the most extreme values for each PC axis: PC1+ = *Exallias brevis*, PC2+ = *Chasmodes bosquianus*, PC1− = *Plagiotremus laudandus*, and PC2− = *Cirripectes castaneus*. The inset panel shows the phylomorphospace with the outlier taxon *Xiphasia setifer* included and indicated with a red arrow. The coloring scheme of the inset panel mirrors that of the main panel.

Phylomorphospace plots reveal that combtooth blennies from all tidal zones exhibit diverse body shapes and substantial overlap in the morphospaces of subtidal, intertidal, and freshwater combtooth blennies (Fig. 5). Intertidal combtooth blennies (41 species) were less morphologically diverse than subtidal combtooth blennies (27 species). Subtidal lineages had significantly higher disparity than intertidal lineages and occupied a region of morphospace three times larger than intertidal lineages (Procrustes variance = 0.009 vs. Procrustes variance = 0.003; *P*-value < 0.001), while other habitat types showed no significant differences in body shape disparity (Supplementary Table S2). Body size disparity did not vary significantly between any tidal zones (Supplementary Table S3). The intertidal combtooth blenny morphospace was nearly completely contained within the subtidal combtooth blenny morphospace (i.e., intertidal combtooth blennies do not exhibit morphological novelty). Subtidal combtooth blenny species represented both extreme ends of PC1 and one of the extreme ends of PC2. The morphospace of the supralittoral combtooth blenny lineage (two species: *Andamia tetradactylus* and *Alticus arnoldorum*) was much smaller than subtidal and intertidal combtooth blenny morphospaces and exhibited limited overlap with the morphospaces of other tidal zones. The supralittoral combtooth blennies in our study possess elongate, eel-like bodies, dorsal placement of the eyes, and inferior placement of the mouth (Fig. 5). Other elongate taxa in our study had either lateral placement of the eyes (e.g., *Plagiotremeus, Xiphasia*) or a more terminal mouth (*Omobranchus banditus*; Figs. 3 and 4). Phylogenetic MANOVA did not find correlations between tidal zone and body shape (full dataset *r*^2^ = 0.04, *P*-value = 0.40; reduced dataset *r*^2^ = 0.05, *P*-value = 0.38) and phylogenetic ANOVA did not find correlations between tidal zone and body size (*P*-value = 0.83). Convergence tests using the full dataset did not find evidence for convergence associated with colonization of intertidal zones (*C*_1_*P*-value = 0.20, C_2_*P*-value = 0.31, C_3_*P*-value = 0.41, C_4_*P*-value = 0.90). Convergence tests using the reduced dataset also did not find evidence for convergence associated with colonization of intertidal zones (*C_1_ P*-value = 0.06, C_2_*P*-value = 0.11, C_3_*P*-value = 0.30, C_4_*P*-value = 0.75).

## Discussion

Subtidal combtooth blennies have significantly higher body shape disparity and occupy a region of morphospace three times larger than intertidal lineages, with many of the species in regions of morphospace unique to subtidal combtooth blennies, exhibiting distinct microhabitat use. The intertidal morphospace was almost entirely contained within the subtidal morphospace, showing that intertidal combtooth blennies did not evolve unique body shapes. We found no significant differences in body size disparity between tidal zones, no correlations between body shape and tidal zone or body size and tidal zone, and no body shape convergence associated with tidal zone. In agreement with previous research, we inferred a subtidal most recent common ancestor of combtooth blennies (Hundt et al. 2014a). Our findings suggest that a subset of combtooth blenny body shapes are suitable for life in both subtidal and intertidal habitats, while some subtidal body shapes, such as blunt, tall, and antero-posteriorly compressed heads with bodies tapering off posteriorly, are selected against in intertidal environments. We found evidence that subtidal environments promote morphological diversification via evolutionary microhabitat transitions, while strong selective pressures in intertidal environments constrain body shape evolution. In addition, environmental filtering may prevent colonization of intertidal zones by certain subtidal body shapes, further contributing to lower intertidal body shape diversity relative to subtidal areas.

### Ecological opportunity promotes subtidal diversity

Subtidal combtooth blennies have significantly higher body shape disparity and occupy a region of morphospace three times larger and almost completely containing intertidal combtooth blennies. Subtidal combtooth blenny species occupying regions of morphospace distinct from the intertidal morphospace belonged to three clades that were not inferred to have descended from intertidal ancestors (i.e., not product of reinvasions of subtidal zones): (1) Plagiotrematinae (*Plagiotremus rhinorhynchos*, *Plagiotremus tapeinosoma*, and *X. setifer*), (2) Williamsichthys (*Exallias brevis*, *Cirripectes castaneus*, *Cirripectes variolosus*, and *Cirripectes polyzona*), and (3) *Ecsenius* (*Ecsenius yaeyamaensis* and *Ecsenius opsifrontalis*). *Xiphasia setifer* and *Plagiotremus* spp. are long, untapered, and have terminal mouths. *Exallias brevis* has a moderately blunt head, is short and highly tapered posteriorly, and has a subterminal mouth and *Cirripectes* spp. and *Ecsenius* spp. have very blunt heads, are short and moderately tapered, have large, subterminal mouths, and eyes positioned anteriorly and dorsally.

All of the morphologically divergent subtidal combtooth blenny clades have ecological niches only found in subtidal zones in our dataset, suggesting that ecological opportunities found in subtidal zones promoted the evolution of unique morphotypes (Smith-Vaniz 1976; Gonçalves and Faria 2009; Hundt et al. 2014b, 2017; Froese and Pauly 2020). The best example of this putative phenomenon is exemplified by members of Plagiotrematinae. *Xiphasia setifer* hides in tube-like invertebrate burrows in sand and mud, whereas most combtooth blennies use hard substrates and structures such as rock crevices, coral, and oyster shells for refuge (Smith-Vaniz 1976; Gonçalves and Faria 2009; Hundt et al. 2014b). Most combtooth blennies are benthic, but plagiotrematinae evolved a pelagic lifestyle (Smith-Vaniz 1976; Hundt et al. 2014b). Several *Plagiotremus* spp. are lepidophagous, and *X. setifer* is one of only a small number of combtooth blennies species that feeds primarily on polychaete worms (Hundt and Simons 2018). *Plagiotremus rhinorhynchos* change their color to mimic cleaner wrasses (*Labroides* spp.), allowing them to approach victims undetected and bite off scales (Smith-Vaniz 1976; Côté and Cheney 2007; Cheney et al. 2009; Hundt et al. 2014b; Hundt and Simons 2018). *Plagiotremus rhinorhynchos* and *P. tapeinosoma* both engage in social mimicry and have evolved color patterns that allow them blend in with schooling fishes such as the Marquesan endemic wrasse (*Coris hewetti*) and the blunthead wrasse (*Thalassoma amblycephalum*), allowing them to join schools and avoid detection by predators (Russell et al. 1976; Smith-Vaniz 1976; Moland et al. 2005; Côté and Cheney 2007; Delrieu‐Trottin et al. 2016). In addition to driving color evolution in combtooth blennies, these mimetic interactions may also exert selective pressure on body shape. The impacts of mimetic relationships on fish body shape evolution are poorly understood, but qualitative reports suggest mimic body shapes typically resemble their mimicry model species (Russell et al. 1976; Moland et al. 2005; Robertson 2013). *Exallias brevis* is a hard coral reef specialist, living almost exclusively among *Acropora* spp., and is the only combtooth blenny with a diet primarily comprised of coral polyps (Carlson 1992, 2012; Hundt et al. 2014b; Hundt and Simons 2018; Froese and Pauly 2020). The three *Cirripectes* and two *Ecsenius* species that we found to have distinct morphologies inhabit shallow, outer coral reef ridges (Springer 1988; Williams 1988; Froese and Pauly 2020). *Exallias brevis* is a hard coral reef specialist, living almost exclusively among *Acropora* spp., and is the only combtooth blenny with a diet primarily comprised of coral polyps (Carlson 1992, 2012; Hundt et al. 2014b; Hundt and Simons 2018; Froese and Pauly 2020). The three *Cirripectes* and two *Ecsenius* species that we found to have distinct morphologies inhabit shallow, outer coral reef ridges (Springer 1988; Williams 1988; Froese and Pauly 2020).

### Evolutionary constraints and environmental filtering limit intertidal diversity

Harsh and dynamic conditions and few ecological opportunities in intertidal environments may have limited body shape diversity in intertidal combtooth blennies. Although intertidal combtooth blennies exhibit differences in substrate preferences, turbulences, and depths within intertidal zones used (La Mesa and Vacchi 2005; Gonçalves and Faria 2009), these microhabitat differences among intertidal blennies did not generate body shape diversity. We suggest this is because strong intertidal selective pressures constrained body shape evolution, preventing substantial deviations of body shape from an adaptive optima (de Alencar et al. 2017). Future studies could determine if differences in intertidal combtooth blenny microhabitat use is associated with evolution of other aspects of morphology, such as fin anatomy (Brandstätter 1990; Horn 1999; Kotrschal 1999). In subtidal combtooth blennies, body shape evolution was associated with use of resources that are not readily available in intertidal zones (e.g., *Acropora* coral and pelagic habitats) and complex mimetic interactions that are not possible in intertidal zones due to their occurrence in the water column and involvement of primarily subtidal species (Russell et al. 1976; Smith-Vaniz 1976; Côté and Cheney 2007; Delrieu‐Trottin et al. 2016). This suggests that limited ecological opportunity in intertidal relative to subtidal zones, due to lower resource diversity, also may have constrained body shape diversification.

Combtooth blennies that successfully colonized intertidal zones were from a restricted region of morphospace (intermediate PC1 and high PC2 values), suggesting that environmental filtering may have played a role in shaping intertidal combtooth blenny body shape diversity. For example, the tall, blunt heads of subtidal *E. brevis*, and especially *Cirripectes* spp. and *Ecsenius* spp., are not found in intertidal species in our dataset. This body shape may subject these taxa to high drag forces, making them poorly suited to fast, turbulent water flows in intertidal environments and preventing them from colonizing intertidal habitats (Langerhans 2008; Wiegleb et al. 2020). This idea is supported by a study finding that tall heads and bodies are associated with lower flows in stream-dwelling banded sculpins (*Cottus carolinae*; Kerfoot and Schaefer 2006), but not by a study reporting that some intertidal sculpin species (Oligocottinae) had blunter heads than subtidal species (Buser et al. 2017). The long and untapered body shapes of *Xiphasia setifer* and *Plagiotremus* spp. are also absent from intertidal zones in our dataset, suggesting these body shapes are incompatible with the intertidal. However, in contradiction with this hypothesis, several lineages of intertidal fishes have body shapes that appear similarly long and untapered, including gunnels (Pholidae), pricklebacks (Stichaeidae), snake eels (Ophichthidae), and graveldivers (Scytalinidae; Horn 1999; Boyle and Horn 2006; Godinho and Lotufo 2010). The similarity of these body shapes may only be superficial, and quantitative comparisons might identify differences in the body shapes of long intertidal fishes and those of *Xiphasia setifer* and *Plagiotremus* spp. Alternatively, environmental filtering may not responsible for the absence of these body shapes in intertidal zones in our dataset.

We found that transitions to intertidal zones did not lead to the evolution of novel body shapes or sizes. Furthermore, we found that lineages that transitioned from intertidal to subtidal environments did not have body shapes or sizes differing substantially from close intertidal relatives. This suggests that a subset combtooth blenny body shapes are suitable for both subtidal and intertidal zones. This might be the result of intertidal ancestry deep in the blenniiform lineage. Hundt et al. (2014a) inferred a subtidal most recent common ancestor of combtooth blennies. However, this subtidal lineage could have arisen from an intertidal blenniiform ancestor and a body shape capable of inhabiting intertidal zones was retained (i.e., are plesiomorphic) in the most recent common ancestor of combtooth blennies and multiple descendant lineages. All six blenniiform families contain intertidal species, which suggests this “early intertidal ancestry hypothesis” is plausible (Muñoz and Ojeda 1997; Fukao 1980; Boyle and Horn 2006; Teixeira et al. 2013). This would explain why we found no evidence of convergent body shape or size evolution associated with transitions between tidal zones. Phylogenetic relationships among major blenniiform lineages remain unclear and must be resolved before the early intertidal ancestry hypothesis can be tested (Lin and Hastings 2013).

### Intertidal combtooth blenny body shapes do not support classic hypotheses

Intertidal combtooth blennies do not have the overtly streamlined or dorsoventrally compressed body shapes, relative to subtidal species, that previous studies predicted would result from adaptation to high intertidal turbulence and water velocity (Horn 1999; Kotrschal 1999; Boyle and Horn 2006; Soares et al. 2013). Instead, we found that intertidal blennies were characterized by somewhat tapered bodies, intermediate body lengths, and moderately blunt and anteroposteriorly compressed heads. These are body shapes that appear to resemble those of intertidal sculpins reported by Buser et al. (2017). It is not entirely unexpected that our study does not support classic hypotheses about fish intertidal fish body shapes because they are primally based on research investigating relationships between fish body shape and water flow in freshwater systems, which may have flow regimes too dissimilar from intertidal conditions to yield relevant predictions about body shape evolution in intertidal zones (Kerfoot and Schaefer 2006; Meyers and Belk 2014; Natsumeda et al. 2014). In addition, many hypotheses about body shape evolution in fishes do not differentiate between pelagic or benthic fishes, even though different associations between water flow and body shape evolution have been found in benthic versus pelagic fishes (Horn 1999; Kotrschal 1999; Boyle and Horn 2006; Langerhans 2008; Soares et al. 2013; de Barros et al. 2019). Finally, the body shapes of benthic fishes exhibit inconsistent associations with water flow, with some studies identifying correlations between streamlining and low flow velocities and others finding the opposite pattern, possibly due to differences in fish activity levels, swimming mode, body size, and substrate use (Kerfoot and Schaefer 2006; Langerhans 2008; Meyers and Belk 2014; Natsumeda et al. 2014; Hopper et al. 2017; Jacobson et al. 2017; Chiarello-Sosa et al. 2018; de Barros et al. 2019).

### Conclusions and future directions

Additional studies of intertidal fish evolution could test for morphological convergence using a larger clade, such as the entire blenniiform order, to sample a larger range of morphologies and additional origins of intertidal habitat use. Furthermore, using lateral photographs limited our ability to consider variation in the left–right axis (*z*-dimension) of our study taxa and thus, potentially precluded our ability to detect some meaningful patterns of combtooth Blenny shape variation (Cardini and Thorington 2006; Álvarez and Perez 2013; Cardini 2014; Buser et al. 2018). Even without full consideration of the *z*-dimension, analyses of 2D images have been shown to capture many of the patterns in shape variation found using 3D approaches, especially in studies that consider questions in broad phylogenetic scopes (McWhinnie and Parsons 2019; Wasiljew et al. 2020; White et al. 2020). Nevertheless, researchers might benefit from using computed tomography data to more comprehensively quantify combtooth blenny body shapes, measure functional consequences of body shape using experimental approaches, and examine other aspects of intertidal fish morphology such as fin, tooth, epidermal, and muscular anatomy (Langerhans 2008; Buser et al. 2018; Cohen and Hernandez 2018; Evans et al. 2019a,b; Buser et al. 2019; Kolmann et al. 2019; Rutledge et al. 2019; Buser et al. 2020; Cohen et al. 2020). It is important to recognize that intertidal habitats are heterogenous and accounting for water flows experienced and microhabitats used by intertidal fishes in comparative analyses, in addition to simple intertidal versus subtidal comparisons, will be important for improving our understanding of intertidal fish evolution.

## Supplementary Material

obab004_Supplementary_Data

## Online supplementary data

Supplementary data available at IOB online.
