# Supplementary material for: Patterns of Body Shape Diversity and Evolution in Intertidal and Subtidal Lineages of Combtooth Blennies (Blenniidae)
Source: Integr Org Biol. 2021 Mar 16;3(1):obab004. doi: 10.1093/iob/obab004 (PMC8077888; doi:10.1093/iob/obab004)
Supplement: obab004_Supplementary_Data [file obab004_supplementary_data.zip › SupplementaryInformation1.09_SupplementaryTables.docx]

**Supplementary Table 1**. Principal component (PC) loadings for the *x* and *y* values of each of the 16 landmarks used to represent lateral blenny body shape in this study.

|  | **PC1** | **PC2** | **PC3** | **PC4** | **PC5** | **PC6** | **PC7** |
| --- | --- | --- | --- | --- | --- | --- | --- |
| **LM1X** | -0.0547086 | 0.18837775 | -0.0033008 | 0.26858256 | -0.671316 | 0.00918057 | -0.2265234 |
| **LM1Y** | -0.315036 | 0.25985638 | -0.1315706 | -0.1525461 | 0.36823683 | 0.13544371 | 0.11854695 |
| **LM2X** | 0.19234669 | 0.01615192 | -0.0350426 | 0.01782977 | 0.05704055 | -0.3024798 | -0.0097202 |
| **LM2Y** | -0.0796704 | 0.02143401 | -0.1022189 | 0.03114248 | 0.11255773 | -0.243627 | 0.04018393 |
| **LM3X** | 0.10953257 | 0.05966256 | 0.03251135 | -0.0525133 | -0.007007 | 0.19058834 | -0.0174495 |
| **LM3Y** | -0.0978154 | 0.01166504 | -0.1087901 | 0.02468188 | 0.0712765 | -0.1674361 | 0.01420072 |
| **LM4X** | 0.0914541 | -0.0212532 | -0.0622452 | -0.0489212 | -0.0635203 | 0.17511241 | -0.0036602 |
| **LM4Y** | -0.0055463 | -0.1916791 | -0.0779806 | 0.05020546 | -0.1740932 | 0.15662961 | -0.0771604 |
| **LM5X** | 0.15560917 | -0.1425786 | -0.1560087 | -0.0215124 | 0.04127445 | -0.07931 | -0.0088649 |
| **LM5Y** | 0.03516498 | -0.1458663 | -0.0365271 | 0.00783913 | -0.1016083 | 0.11689858 | -0.1048173 |
| **LM6X** | -0.4032382 | -0.0342175 | 0.59855771 | 0.54407232 | 0.1957089 | 0.08172779 | 0.13115881 |
| **LM6Y** | 0.30951412 | 0.13829126 | 0.34162039 | -0.0117843 | -0.1367639 | 0.1705664 | 0.16904496 |
| **LM7X** | -0.2955982 | 0.03921971 | 0.08088103 | -0.2601557 | -0.0784479 | -0.1268518 | -0.2577005 |
| **LM7Y** | 0.03904569 | 0.27548535 | 0.08701444 | -0.1508616 | 0.15914052 | 0.17576826 | -0.1268974 |
| **LM8X** | -0.2869957 | 0.12950603 | 0.16272128 | -0.4024488 | -0.0562695 | -0.2436067 | -0.345877 |
| **LM8Y** | 0.25106171 | 0.15849521 | 0.27495117 | -0.1508093 | 0.04420243 | -0.0189562 | -0.1814976 |
| **LM9X** | -0.0630668 | 0.04542936 | 0.11612943 | -0.4234014 | -0.2750875 | 0.0050361 | 0.73247821 |
| **LM9Y** | 0.30185773 | 0.10233756 | 0.22062545 | 0.00015937 | 0.10122856 | 0.05931403 | -0.1666238 |
| **LM10X** | 0.2200937 | 0.21618351 | -0.105923 | 0.12017841 | 0.09562114 | 0.11332087 | 0.06557228 |
| **LM10Y** | 0.07131655 | -0.2458724 | 0.07145089 | 0.07931622 | 0.00189406 | -0.4311259 | 0.11448477 |
| **LM11X** | 0.0224528 | 0.3968369 | -0.3028175 | 0.2826826 | 0.0854524 | -0.2598129 | 0.06139125 |
| **LM11Y** | 0.08902775 | -0.0997194 | 0.0756299 | -0.0165086 | -0.0003645 | -0.1814128 | -0.0506702 |
| **LM12X** | 0.21869236 | 0.14053654 | -0.1147538 | 0.13061121 | 0.13558711 | -0.1237663 | 0.10927298 |
| **LM12Y** | -0.0106723 | -0.3007869 | 0.08206193 | 0.03450474 | -0.0871917 | -0.2874553 | 0.10605776 |
| **LM13X** | 0.07754177 | -0.2920153 | -0.0905271 | -0.0200095 | 0.06823599 | 0.15983932 | -0.0661379 |
| **LM13Y** | -0.1391309 | -0.0168614 | -0.1653711 | 0.05807541 | -0.0934358 | 0.1529697 | 0.01186751 |
| **LM14X** | -0.036431 | -0.2260385 | -0.0360463 | -0.0541686 | 0.20768761 | 0.10633762 | -0.048415 |
| **LM14Y** | -0.1530505 | 0.01715371 | -0.1796693 | 0.05856179 | -0.0675901 | 0.12546611 | 0.06823384 |
| **LM15X** | 0.02440129 | -0.2511094 | -0.0283323 | -0.0439665 | 0.13821546 | 0.147583 | -0.0758501 |
| **LM15Y** | -0.0933281 | -0.0493786 | -0.195464 | 0.09909407 | -0.1642159 | 0.13638447 | -0.0017195 |
| **LM16X** | 0.02791401 | -0.2646917 | -0.0558034 | -0.0368595 | 0.12682467 | 0.14710151 | -0.0396749 |
| **LM16Y** | -0.2027386 | 0.06544562 | -0.1557625 | 0.03892924 | -0.0332732 | 0.10057228 | 0.0667658 |
|  | **PC8** | **PC9** | **PC10** | **PC11** | **PC12** | **PC13** | **PC14** |
| **LM1X** | 0.41096331 | -0.252074 | 0.04699741 | -0.1677077 | 0.02736137 | 0.03167328 | -0.0830143 |
| **LM1Y** | 0.32581633 | -0.1652492 | 0.48232516 | -0.0367727 | -0.1086348 | 0.21330057 | -0.3047389 |
| **LM2X** | 0.16615136 | 0.11756089 | -0.247546 | 0.21649253 | 0.27235588 | 0.5486697 | -0.1452061 |
| **LM2Y** | 0.03750879 | -0.2576218 | -0.1880846 | -0.126892 | 0.22283321 | -0.0387303 | -0.0685465 |
| **LM3X** | -0.1746897 | -0.3567954 | 0.01694639 | 0.10521284 | 0.00157739 | 0.09872097 | 0.18182436 |
| **LM3Y** | -0.0268014 | -0.2757644 | -0.1714801 | -0.0908801 | 0.1320331 | -0.2970985 | -0.1024617 |
| **LM4X** | -0.2520442 | -0.2795219 | 0.14562468 | 0.04197075 | -0.2933739 | -0.2067141 | 0.05054789 |
| **LM4Y** | -0.0079564 | 0.12124961 | 0.11603568 | 0.18388285 | -0.3177041 | 0.17811757 | 0.13929269 |
| **LM5X** | 0.26039331 | 0.51655135 | 0.15599069 | -0.3264393 | 0.03459008 | -0.4123446 | -0.0553631 |
| **LM5Y** | 0.05324795 | 0.28116905 | 0.09762255 | 0.05316035 | -0.146402 | 0.25909606 | 0.04525846 |
| **LM6X** | -0.0174943 | 0.12391565 | -0.1015483 | 0.02603983 | -0.0803922 | -0.0498428 | -0.0580327 |
| **LM6Y** | -0.1902667 | 0.02308878 | 0.377635 | -0.0970126 | 0.55262666 | 0.01412536 | 0.07162136 |
| **LM7X** | -0.241983 | 0.13180218 | 0.18274768 | -0.0063325 | 0.1511324 | 0.07735128 | 0.10734104 |
| **LM7Y** | 0.10180038 | 0.10883271 | -0.2703965 | 0.23935325 | 0.11360096 | -0.1346359 | 0.26806287 |
| **LM8X** | -0.2211491 | 0.10633547 | -0.0049583 | -0.0532471 | -0.0566382 | -0.0361873 | -0.1208898 |
| **LM8Y** | 0.22985906 | -0.0143589 | -0.2213845 | 0.13146422 | -0.3132023 | -0.0717862 | -0.0114477 |
| **LM9X** | 0.14195626 | 0.05379841 | -0.1751878 | 0.06181416 | -0.1214742 | -0.0646589 | -0.0298984 |
| **LM9Y** | 0.08227211 | 0.03428583 | 0.06781366 | -0.1041126 | -0.0696695 | -0.1332156 | -0.1897062 |
| **LM10X** | -0.2134219 | 0.10814561 | -0.084568 | -0.1726669 | -0.1319898 | 0.20290241 | -0.1198498 |
| **LM10Y** | -0.0490019 | -0.1122027 | 0.17707075 | -0.1038555 | -0.1284575 | 0.08056135 | 0.11695172 |
| **LM11X** | 0.03650136 | 0.08732755 | 0.19559163 | 0.42517738 | -0.0008186 | -0.2523269 | 0.29259266 |
| **LM11Y** | -0.0631572 | -0.1338423 | -0.1491237 | -0.1717762 | -0.157693 | -0.05674 | 0.03083823 |
| **LM12X** | -0.3002796 | 0.01162661 | -0.0212658 | -0.2596891 | -0.2125291 | 0.14169099 | -0.1181771 |
| **LM12Y** | -0.0266221 | -0.0089179 | 0.22289018 | 0.30319031 | -0.014704 | -0.0327321 | 0.04928529 |
| **LM13X** | -0.0013713 | -0.0590794 | -0.0027632 | 0.26125235 | 0.11646484 | -0.1091389 | -0.3057349 |
| **LM13Y** | -0.1330874 | 0.09558831 | -0.13781 | -0.0037077 | 0.06912595 | -0.0239167 | -0.0196519 |
| **LM14X** | 0.18814274 | -0.1099461 | -0.0407558 | -0.2049723 | 0.09492828 | 0.06622926 | 0.42945966 |
| **LM14Y** | -0.1379445 | 0.11703707 | -0.1188057 | -0.1169099 | 0.0945831 | -0.0145137 | 0.03626956 |
| **LM15X** | 0.15676669 | -0.0857145 | -0.0367458 | 0.06420713 | 0.11531016 | -0.0399972 | -0.1088503 |
| **LM15Y** | -0.2254851 | 0.09336499 | -0.1384586 | 0.19501516 | 0.08440437 | -0.0935241 | -0.3689472 |
| **LM16X** | 0.0615582 | -0.1139325 | -0.0285595 | -0.011112 | 0.08349567 | 0.00397281 | 0.08325089 |
| **LM16Y** | 0.02981819 | 0.09334083 | -0.1458493 | -0.2541468 | -0.0127402 | 0.15169217 | 0.30792003 |
|  | **PC15** | **PC16** | **PC17** | **PC18** | **PC19** | **PC20** | **PC21** |
| **LM1X** | -0.0198741 | 0.14613619 | 0.08368623 | -0.0354025 | -0.0313239 | -0.005982 | 0.0329478 |
| **LM1Y** | -0.1185123 | 0.03753344 | -0.0326252 | -0.0292973 | -0.0620351 | 0.00349352 | 0.01340261 |
| **LM2X** | 0.04786286 | -0.1386181 | -0.0654068 | -0.2421679 | -0.0381019 | 0.05369489 | 0.03206114 |
| **LM2Y** | 0.03694912 | 0.12090306 | 0.06431097 | 0.07907002 | 0.13253229 | 0.10930779 | -0.3500262 |
| **LM3X** | -0.0573068 | 0.03877819 | 0.03557564 | 0.53904395 | 0.21056188 | -0.2215611 | 0.05772446 |
| **LM3Y** | 0.16374838 | -0.0603614 | -0.0629367 | 0.03086309 | -0.2185921 | -0.0888258 | 0.26362173 |
| **LM4X** | 0.1133195 | -0.0289731 | 0.02696799 | -0.4241994 | -0.1166287 | 0.14159704 | -0.0153786 |
| **LM4Y** | -0.0582524 | -0.0505959 | -0.0146131 | -0.1714003 | -0.064786 | 0.24485965 | -0.1739967 |
| **LM5X** | -0.1131377 | 0.10908169 | 0.01723064 | 0.13627278 | -0.0567128 | 0.02906829 | -0.0695523 |
| **LM5Y** | -0.0354663 | 0.04029975 | -0.0024742 | 0.13027536 | 0.21523263 | -0.3649085 | 0.28378281 |
| **LM6X** | -0.0325683 | 0.00660376 | -0.017681 | -0.0171971 | 0.02022292 | -0.0060037 | -0.0134635 |
| **LM6Y** | -0.2235318 | -0.1525334 | 0.0111772 | -0.0592529 | -0.0794731 | 0.0998436 | -0.0791721 |
| **LM7X** | 0.27036356 | 0.33664105 | -0.5462447 | 0.08086329 | -0.0421095 | 0.10568019 | -0.0172218 |
| **LM7Y** | -0.0860661 | 0.55902006 | 0.2887212 | -0.2431179 | -0.0392125 | -0.0240045 | 0.14253028 |
| **LM8X** | -0.182962 | -0.3343471 | 0.44275122 | -0.059559 | 0.05076552 | -0.0775173 | -0.0232942 |
| **LM8Y** | -0.1190737 | -0.1380098 | -0.2122844 | 0.34719113 | -0.1181739 | 0.33277395 | -0.2059865 |
| **LM9X** | 0.08228815 | -0.0033654 | -0.0681009 | -0.0294236 | 0.10642128 | -0.0442096 | 0.05415506 |
| **LM9Y** | 0.59105411 | -0.1849702 | -0.0197584 | -0.15124 | 0.34548235 | -0.1536822 | 0.09927317 |
| **LM10X** | 0.16569936 | 0.10237902 | 0.03603489 | 0.06382685 | -0.5203659 | -0.1934822 | -0.0481065 |
| **LM10Y** | -0.0391 | 0.27320837 | 0.0831719 | -0.0717661 | 0.26431848 | 0.13690973 | 0.09669248 |
| **LM11X** | -0.0546259 | -0.2422236 | -0.1290438 | -0.0130054 | 0.13852336 | -0.0887014 | -0.0066614 |
| **LM11Y** | -0.4386521 | -0.0656814 | -0.4045664 | -0.2145546 | -0.1366978 | -0.4191366 | 0.02879365 |
| **LM12X** | -0.1130757 | 0.18216235 | 0.15348799 | 0.08486177 | 0.1980809 | 0.2523404 | 0.06465872 |
| **LM12Y** | 0.23333266 | 0.01921613 | 0.30315068 | 0.20666248 | -0.4256208 | -0.1037032 | -0.026173 |
| **LM13X** | -0.1042248 | 0.13737869 | 0.01629649 | 0.02852045 | 0.13988681 | -0.1932335 | -0.3662519 |
| **LM13Y** | 0.17769608 | -0.0907932 | -0.0968641 | 0.00186676 | 0.09852096 | 0.12493332 | -0.2195186 |
| **LM14X** | 0.00811025 | -0.239641 | 0.0008761 | 0.02070215 | -0.0536596 | 0.26043757 | 0.30474697 |
| **LM14Y** | -0.078272 | -0.0762258 | -0.0544586 | -0.0257084 | 0.02145704 | -0.0682223 | 0.07876176 |
| **LM15X** | 0.02218483 | -0.0585176 | 0.02945383 | 0.08944654 | -0.112648 | 0.09505721 | 0.22892395 |
| **LM15Y** | -0.1519737 | -0.0663794 | -0.0243548 | 0.08813271 | 0.06094714 | 0.27940245 | 0.34411893 |
| **LM16X** | -0.0320532 | -0.013475 | -0.0158839 | -0.2225828 | 0.10708768 | -0.1071848 | -0.2152878 |
| **LM16Y** | 0.14612 | -0.1646304 | 0.17440378 | 0.08227588 | 0.00610042 | -0.109041 | -0.2961044 |
|  | **PC15** | **PC16** | **PC17** | **PC18** | **PC19** | **PC20** | **PC21** |
| **LM1X** | -0.0198741 | 0.14613619 | 0.08368623 | -0.0354025 | -0.0313239 | -0.005982 | 0.0329478 |
| **LM1Y** | -0.1185123 | 0.03753344 | -0.0326252 | -0.0292973 | -0.0620351 | 0.00349352 | 0.01340261 |
| **LM2X** | 0.04786286 | -0.1386181 | -0.0654068 | -0.2421679 | -0.0381019 | 0.05369489 | 0.03206114 |
| **LM2Y** | 0.03694912 | 0.12090306 | 0.06431097 | 0.07907002 | 0.13253229 | 0.10930779 | -0.3500262 |
| **LM3X** | -0.0573068 | 0.03877819 | 0.03557564 | 0.53904395 | 0.21056188 | -0.2215611 | 0.05772446 |
| **LM3Y** | 0.16374838 | -0.0603614 | -0.0629367 | 0.03086309 | -0.2185921 | -0.0888258 | 0.26362173 |
| **LM4X** | 0.1133195 | -0.0289731 | 0.02696799 | -0.4241994 | -0.1166287 | 0.14159704 | -0.0153786 |
| **LM4Y** | -0.0582524 | -0.0505959 | -0.0146131 | -0.1714003 | -0.064786 | 0.24485965 | -0.1739967 |
| **LM5X** | -0.1131377 | 0.10908169 | 0.01723064 | 0.13627278 | -0.0567128 | 0.02906829 | -0.0695523 |
| **LM5Y** | -0.0354663 | 0.04029975 | -0.0024742 | 0.13027536 | 0.21523263 | -0.3649085 | 0.28378281 |
| **LM6X** | -0.0325683 | 0.00660376 | -0.017681 | -0.0171971 | 0.02022292 | -0.0060037 | -0.0134635 |
| **LM6Y** | -0.2235318 | -0.1525334 | 0.0111772 | -0.0592529 | -0.0794731 | 0.0998436 | -0.0791721 |
| **LM7X** | 0.27036356 | 0.33664105 | -0.5462447 | 0.08086329 | -0.0421095 | 0.10568019 | -0.0172218 |
| **LM7Y** | -0.0860661 | 0.55902006 | 0.2887212 | -0.2431179 | -0.0392125 | -0.0240045 | 0.14253028 |
| **LM8X** | -0.182962 | -0.3343471 | 0.44275122 | -0.059559 | 0.05076552 | -0.0775173 | -0.0232942 |
| **LM8Y** | -0.1190737 | -0.1380098 | -0.2122844 | 0.34719113 | -0.1181739 | 0.33277395 | -0.2059865 |
| **LM9X** | 0.08228815 | -0.0033654 | -0.0681009 | -0.0294236 | 0.10642128 | -0.0442096 | 0.05415506 |
| **LM9Y** | 0.59105411 | -0.1849702 | -0.0197584 | -0.15124 | 0.34548235 | -0.1536822 | 0.09927317 |
| **LM10X** | 0.16569936 | 0.10237902 | 0.03603489 | 0.06382685 | -0.5203659 | -0.1934822 | -0.0481065 |
| **LM10Y** | -0.0391 | 0.27320837 | 0.0831719 | -0.0717661 | 0.26431848 | 0.13690973 | 0.09669248 |
| **LM11X** | -0.0546259 | -0.2422236 | -0.1290438 | -0.0130054 | 0.13852336 | -0.0887014 | -0.0066614 |
| **LM11Y** | -0.4386521 | -0.0656814 | -0.4045664 | -0.2145546 | -0.1366978 | -0.4191366 | 0.02879365 |
| **LM12X** | -0.1130757 | 0.18216235 | 0.15348799 | 0.08486177 | 0.1980809 | 0.2523404 | 0.06465872 |
| **LM12Y** | 0.23333266 | 0.01921613 | 0.30315068 | 0.20666248 | -0.4256208 | -0.1037032 | -0.026173 |
| **LM13X** | -0.1042248 | 0.13737869 | 0.01629649 | 0.02852045 | 0.13988681 | -0.1932335 | -0.3662519 |
| **LM13Y** | 0.17769608 | -0.0907932 | -0.0968641 | 0.00186676 | 0.09852096 | 0.12493332 | -0.2195186 |
| **LM14X** | 0.00811025 | -0.239641 | 0.0008761 | 0.02070215 | -0.0536596 | 0.26043757 | 0.30474697 |
| **LM14Y** | -0.078272 | -0.0762258 | -0.0544586 | -0.0257084 | 0.02145704 | -0.0682223 | 0.07876176 |
| **LM15X** | 0.02218483 | -0.0585176 | 0.02945383 | 0.08944654 | -0.112648 | 0.09505721 | 0.22892395 |
| **LM15Y** | -0.1519737 | -0.0663794 | -0.0243548 | 0.08813271 | 0.06094714 | 0.27940245 | 0.34411893 |
| **LM16X** | -0.0320532 | -0.013475 | -0.0158839 | -0.2225828 | 0.10708768 | -0.1071848 | -0.2152878 |
| **LM16Y** | 0.14612 | -0.1646304 | 0.17440378 | 0.08227588 | 0.00610042 | -0.109041 | -0.2961044 |
|  | **PC22** | **PC23** | **PC24** | **PC25** | **PC26** | **PC27** | **PC28** |
| **LM1X** | 0.02341597 | 0.01960563 | 0.02792992 | 0.03319634 | 0.0161591 | -0.0103541 | -0.0526519 |
| **LM1Y** | 0.01684403 | -0.0090043 | 0.04019982 | 0.04662607 | 0.01603331 | 0.03576343 | -0.0456934 |
| **LM2X** | -0.0272654 | 0.00933668 | -0.047016 | 0.1073256 | -0.0213262 | -0.0142477 | -0.0436614 |
| **LM2Y** | 0.29069994 | 0.07868276 | 0.1567456 | -0.4467746 | 0.00722434 | -0.1358103 | 0.0877215 |
| **LM3X** | 0.03437093 | -0.2284294 | 0.14591097 | 0.10084801 | 0.04647074 | 0.02000122 | 0.10637228 |
| **LM3Y** | -0.2909539 | -0.018504 | -0.1110689 | 0.42513197 | -0.0337222 | 0.16235065 | -0.0889525 |
| **LM4X** | -0.0441319 | 0.28654761 | -0.1729565 | -0.2346057 | -0.03476 | -0.0653734 | -0.0980536 |
| **LM4Y** | 0.11812155 | -0.4326345 | 0.10277958 | 0.2350743 | -0.0538378 | -0.0751422 | 0.17995699 |
| **LM5X** | 0.03498671 | -0.06966 | 0.06513341 | 0.0336336 | 0.01425288 | 0.05466407 | 0.04006394 |
| **LM5Y** | -0.0736339 | 0.37741861 | -0.0977519 | -0.2332987 | 0.10440877 | 0.06745761 | -0.1871361 |
| **LM6X** | -0.0005278 | 0.00527144 | 0.00359945 | -0.004854 | -0.0075702 | 0.00663611 | -0.0101275 |
| **LM6Y** | -0.0558595 | 0.02392126 | -0.0946782 | 0.00324973 | 0.01007228 | 0.04263122 | -0.0137221 |
| **LM7X** | 0.0694734 | 0.05749558 | -0.0726622 | 0.03419738 | -0.0885592 | -0.0065806 | 0.06616374 |
| **LM7Y** | 0.06423724 | -0.0691 | -0.010046 | 0.03489173 | 0.02350422 | 0.02718854 | -0.0323198 |
| **LM8X** | -0.0811806 | -0.0912882 | 0.07726639 | -0.0363525 | 0.05217664 | 0.00490031 | -0.0080636 |
| **LM8Y** | -0.2150257 | 0.26911583 | -0.0057955 | 0.01132741 | -0.0109334 | -0.0333734 | -0.0209504 |
| **LM9X** | 0.03779546 | -0.0454838 | 0.02524535 | -0.0201327 | -0.0113563 | 0.0055791 | 0.00594417 |
| **LM9Y** | 0.18302582 | -0.1445761 | 0.09019418 | 0.03252103 | -0.1107694 | -0.0713224 | 0.0852667 |
| **LM10X** | -0.2217818 | -0.1530334 | 0.25033611 | -0.2749468 | -0.056386 | 0.0720878 | 0.06844257 |
| **LM10Y** | -0.5169001 | -0.1771149 | 0.03473528 | -0.1536961 | -0.0426367 | 0.04655707 | 0.09368472 |
| **LM11X** | -0.0241139 | -0.0145147 | -0.0243119 | -0.0818887 | 0.01951034 | -0.0011617 | 0.051603 |
| **LM11Y** | 0.31176417 | -0.163062 | -0.0854105 | -0.0527646 | 0.04525006 | 0.04597105 | -0.021645 |
| **LM12X** | 0.3063056 | 0.15737676 | -0.2099337 | 0.35535016 | 0.11244371 | -0.0471231 | -0.1373475 |
| **LM12Y** | 0.31430933 | 0.17567411 | 0.06785955 | 0.10424257 | 0.06297311 | -0.0522698 | -0.0860235 |
| **LM13X** | -0.1416476 | -0.1009224 | -0.1606199 | 0.05058115 | -0.3934887 | -0.1002954 | -0.3496038 |
| **LM13Y** | -0.0869655 | -0.2010662 | 0.03792008 | -0.0514838 | 0.59252211 | 0.19029696 | -0.4432149 |
| **LM14X** | 0.12758796 | -0.0602694 | 0.20803454 | -0.1184739 | -0.2502416 | 0.03034365 | -0.3765604 |
| **LM14Y** | -0.17435 | 0.17705579 | 0.24807516 | 0.1675683 | -0.0328321 | -0.7541346 | 0.05547348 |
| **LM15X** | -0.0237361 | -0.1657471 | -0.4721693 | -0.1974861 | 0.38648417 | -0.2556394 | 0.3628274 |
| **LM15Y** | 0.13691649 | 0.05188101 | 0.11446405 | -0.160023 | -0.2763385 | 0.32350245 | 0.22293908 |
| **LM16X** | -0.0695508 | 0.39371487 | 0.35621328 | 0.25360813 | 0.21619052 | 0.30656319 | 0.37465266 |
| **LM16Y** | -0.02223 | 0.06131249 | -0.4882223 | 0.03740778 | -0.3009181 | 0.18033375 | 0.21461518 |

**Supplementary Table 2.** Pairwise differences between body shape variances (Procrustes variances of landmark coordinates) for each tidal zone. Asterisks represent significant differences at P < 0.05.

|  | **Freshwater** | **Intertidal** | **Subtidal** | **Supralittoral** |
| --- | --- | --- | --- | --- |
| **Freshwater** | - | 5.93 x 10^-4^ | 5.34 x 10^-3^ | 5.41 x 10^-3^ |
| **Intertidal** | 5.93 x 10^-4^ | - | 5.93 x 10^-3^* | 5.99 x 10^-3^ |
| **Subtidal** | 5.34 x 10^-3^ | 5.93 x 10^-3^* | - | 6.13 x 10^-5^ |
| **Supralittoral** | 5.41 x 10^-3^ | 5.99 x 10^-3^ | 6.13 x 10^-5^ | - |

**Supplementary Table 3.** Pairwise differences between body size (maximum reported standard length in cm for each species) variances for each tidal zone. Asterisks represent significant differences at P < 0.05.

|  | **Freshwater** | **Intertidal** | **Subtidal** | **Supralittoral** |
| --- | --- | --- | --- | --- |
| **Freshwater** | - | 7.95 | 70.29 | 8.27 |
| **Intertidal** | 7.95 | - | 62.34 | 16.22 |
| **Subtidal** | 70.29 | 62.34 | - | 78.56 |
| **Supralittoral** | 8.27 | 16.22 | 78.56 | - |
